# Executive function, limbic circuit dynamics and repetitive and restricted behaviors in children with autism spectrum disorder

**DOI:** 10.3389/fnins.2024.1508077

**Published:** 2025-01-15

**Authors:** Xiangyu Zheng, Xinyue Wang, Ruochen Song, Junbin Tian, Li Yang

**Affiliations:** ^1^Peking University Sixth Hospital, Peking University Institute of Mental Health, National Clinical Research Center for Mental Disorders (Peking University Sixth Hospital), NHC Key Laboratory of Mental Health (Peking University), Beijing, China; ^2^Peking University Health Science Center (Peking University), Beijing, China

**Keywords:** executive function, time-varying functional connectivity, repetitive and restricted behaviors, orbitofrontal cortex, autism spectrum disorder

## Abstract

**Objective:**

Repetitive and restricted behaviors (RRBs) are a core symptom of autism spectrum disorder (ASD), but effective treatment approaches are still lacking. Executive function (EF) has been identified as a promising target, as research increasingly shows a link between EF deficits and the occurrence of RRBs. However, the neural mechanisms that connect the two remain unclear. Since the orbitofrontal cortex (OFC) plays a role in both EF and RRBs, its functional connectivity dynamics could offer valuable insights into this relationship.

**Methods:**

This study analyzed data from the Autism Brain Imaging Data Exchange (ABIDE) II database to explore brain function in 93 boys with ASD and 110 typically developing (TD) boys. Time-varying functional connectivity was analyzed between eight OFC subregions and other brain areas. By employing linear regression, the study assessed how atypical connectivity dynamics and EF influence RRBs. Additionally, mediation analysis with bootstrapping was used to determine how EF mediates the relationship between atypical connectivity and RRBs.

**Results:**

We found significant differences in the variance of FC between ASD and TD groups, specifically in the OFC subregion in L-prefrontal and the left amygdala (*t* = 5.00, FDR *q* < 0.01). Regression analyses revealed that increased variance of this FC and EF significantly impacted RRBs, with inhibition, emotional control, and monitor showing strong associations (standardized *β* = 0.60 to 0.62, *p* < 0.01), which also had significant indirect effects on the relationship between the above dynamic FC and RRBs, which accounted for 59% of the total effect.

**Conclusion:**

This study highlights the critical role of EFs as a key mechanism in addressing RRBs in ASD. Specifically, it points out that EFs mediate the influence of atypical time-varying interactions within the OFC-amygdala circuit on RRBs.

## Introduction

Repetitive and restricted behaviors (RRBs) include stereotyped or repetitive motor movements, insistence on sameness, highly restricted interests, and hyper- or hypo-reactivity to sensory input (American Psychiatric Association, 2013). Recent research has highlighted the potential role of executive functions (EF) in understanding RRBs within ASD populations (Jiujias et al., 2017). EF include high-level cognitive processes such as planning, organization, task management, impulse control, and the flexible adaptation to new information (Jones et al., 2018). Its main objective is to produce behavior that is coordinated, orderly, and purposeful. Deficits in EF are frequently observed in individuals with RRB. The previous meta-analyses found that high levels of RRBs related to poor performance on set-shifting and inhibitory control tasks (Iversen and Lewis, 2021). However, the relationship between EF and RRBs is complex and multifaceted, warranting further exploration.

The neural basis of EF is closely tied to several brain regions, especially the orbitofrontal cortex (OFC) (Li et al., 2024; Rudebeck and Rich, 2018). This region is central to EFs and plays a crucial role in emotional regulation, social cognition, and behavioral control (Rudebeck and Rich, 2018). Dysfunction in the OFC can exacerbate the behavioral traits seen in ASD (Liu et al., 2020). Previous research has found that increased volume of the OFC is associated with an increase in RRBs (Hegarty et al., 2020). Blocking the signal transduction of the 5-HT(2A) receptor in OFC results in an increase in these behaviors (Amodeo et al., 2017). In many tasks that indirectly represent repetitive stereotyped behaviors through response inhibition, the OFC shows heightened activity (Liu et al., 2020; Agam et al., 2010). However, these studies focus primarily on structural and genetic aspects, and while there are indirect task-related connections, the functional relationship between the OFC and RRBs remains unclear. Additionally, the role of executive functions EF in this process still requires further exploration.

The current cognitive model hypothesizes that due to poor cognitive control, the brain is in a hyper-sensitive state, which contribute to increased attention to negative information and, consequently, an increase in RRBs (Iversen and Lewis, 2021). The brain’s hyper-sensitive state primarily refers to an enhanced response to external stimuli (Leekam et al., 2011), and the temporal characteristics of brain activity reflect the brain’s response to these stimuli (Northoff and Huang, 2017). Studying the temporal characteristics of functional connectivity between the OFC and related brain regions can help us understand the state of the OFC and associated regions in response to external stimuli (Dinstein et al., 2015). The time-varying characteristics of functional connectivity refer to the dynamic changes in connection strength between different brain regions over time (Allen et al., 2014). Research has shown that individuals with ASD exhibit increased time-varying characteristics of functional connectivity, which may be associated with their behavioral abnormalities (Li et al., 2020). Thus, we hypothesize that the time-varying characteristics of brain regions may increase, indicating greater hypersensitivity and potentially leading to RRBs.

The different subregions of the OFC represent distinct cognitive functions, which can help us gain a more precise understanding of its underlying mechanisms (Qiu et al., 2024). In the study by Evans et al. (2004), it is noted that the medial OFC is involved in regulating motivation, as it connects with the VTA and nucleus accumbens. The lateral OFC, connected to the caudate nucleus, plays a role in motor coordination. Both OFC-striatal pathways communicate with the thalamus and influence motivation and behavior through feedback to the frontal and motor cortex. Additionally, the OFC is linked to the anterior cingulate cortex (ACC), which is involved in executive functions. Understanding the functions of these subregions and their roles in high-functioning ASD can provide crucial neurological insights into the relationship between RRBs and EF (Iversen and Lewis, 2021). Additionally, identifying the specific functional differences among these subregions can inform the development of more targeted intervention strategies to help individuals with high-functioning ASD improve EF and reduce RRBs. Investigating how OFC influences the relationship between EF and RRBs is vital for uncovering ASD’s neurobiological mechanisms.

Given the central role of RRBs in ASD and the potential influence of EF, this study seeks to explore the dynamic relationship between the OFC and RRBs in children with ASD, with a focus on EF as a mediating factor. We will evaluate the time-varying functional connectivity of the OFC in conjunction with comprehensive EF assessments and RRB behavioral evaluations. By analyzing how EF mediates the relationship between OFC connectivity and RRB severity, this study aims to uncover the cognitive and neural mechanisms driving RRBs. Our findings could provide insights for developing targeted interventions to improve EF and reduce RRBs in children with ASD.

## Methods

### Participants

Using the Autism Brain Imaging Data Exchange (ABIDE) II database, this study analyzed 93 boys with ASD and 110 boys with typical developmental, aged 5–13 years, from eight independent sites. There was no significant difference in age [*t* (202) = 1.650, *p* = 0.101] between the children with ASD (mean age = 9.85 ± 2.4) and those with TDs (mean age = 10.09 ± 1.98). This database employs the ADOS-2 for diagnosis, with the ADI-R used as a supplementary assessment tool.

**Figure 1:**
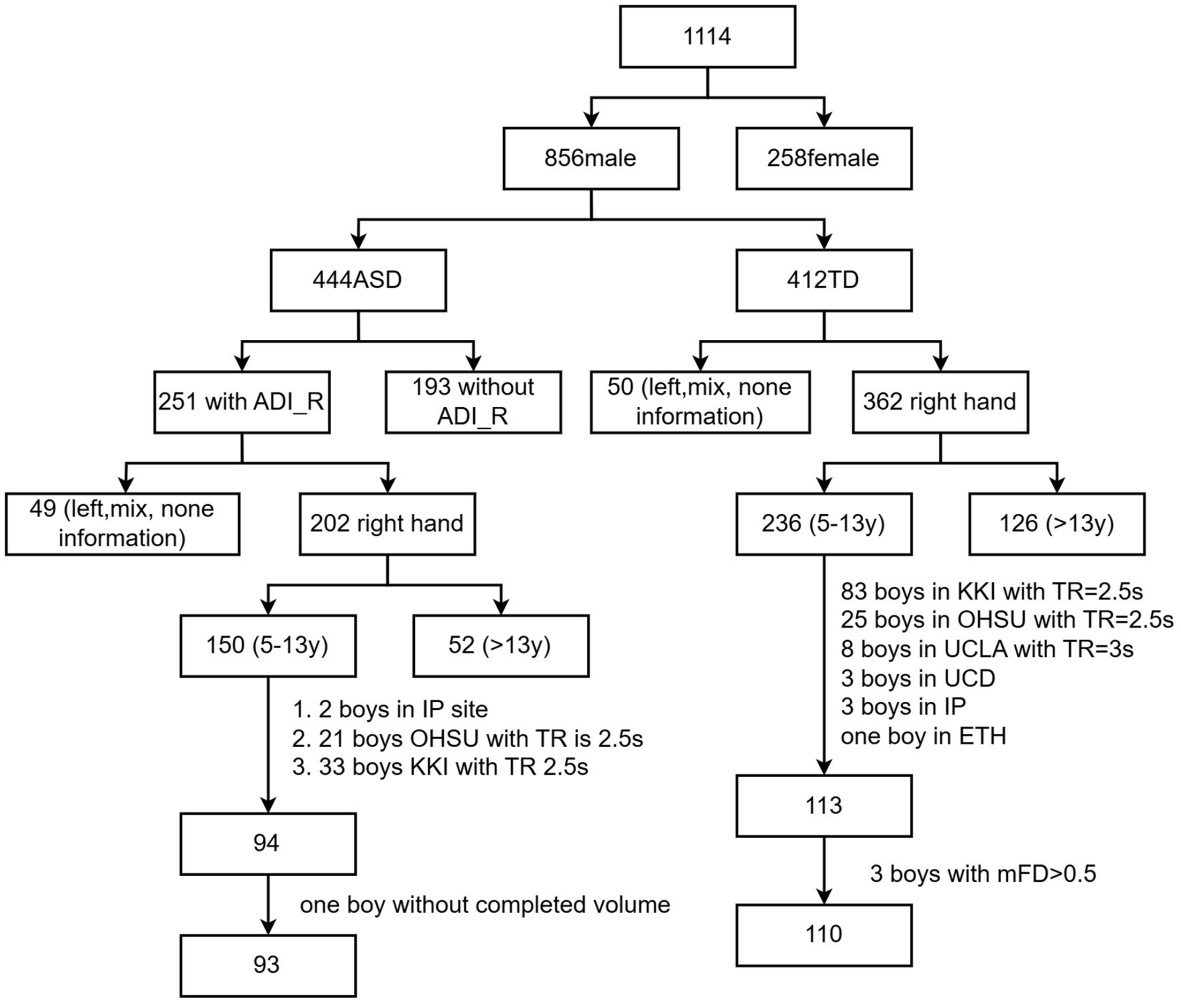
Inclusion process and flow of participants.

Two versions of diagnostic tools, i.e., the Autism Diagnostic Observation Schedule, First Edition (ADOS I) and Autism Diagnostic Observation Schedule, Second Edition (ADOS II), were used in the two-stage ABIDE database, while ADOS_II was found to be more reliable than ADOS_I (Medda et al., 2019); only ABIDE II was used in this study. Only 122 boys with ASD had Autism Diagnostic Interview-Revised (ADI_R) scores. Participants were included when they met the following conditions: (1) boys, as 94% of participants were boys; (2) no severe structural damage in the T1 images; (3) single-band fMRI data; (4) longer than 5 min for scans; (5) considering the challenges of MRI data collection in children with ASD and referencing previous studies (Xie et al., 2022), our head motion criteria were set as follows: the maximum head motion should be less than 5 mm and 5 degrees, and the mean framewise displacement (FD) should be less than 0.5 mm. Specifically, mean FD was computed as the average of the frame-to-frame head displacement across the time series (Power et al., 2012). (6) Full-brain coverage and successful spatial normalization; (7) full-scale IQ higher than 70; (8) sites with more than 5 people. To ensure that each site had an adequate sample size, we removed sites with fewer than five participants, aiming to guarantee the statistical effect and reliability of the analysis results. The participant inclusion process is shown in Figure 1. Information for each site is provided in Table 1.

**Table 1:** Enrollment numbers of ASD and TD groups at each site.

Site	Number of boys	
	ASD	HC
ABIDEII-GU_1	30	24
ABIDEII-NYU_1	29	27
ABIDEII-NYU_2	14	0
ABIDEII-SDSU_1	14	11
ABIDEII-TCD_1	6	8
ABIDEII-EMC_1	15	0
ABIDEII-SU_2	0	16
ABIDEII-U_MIA_1	0	9

### Diagnosis

The Autism Diagnostic Interview-Revised (ADI-R) is a structured interview used for diagnosing autism and planning treatment (Lord et al., 1994). Conducted by a trained psychologist in a quiet setting, the interview lasts one to 2 h and involves caregivers answering 93 questions across three sections: social interaction, communication and language, repetitive and restricted behaviors.

### Executive function

The Behavior Rating Inventory of Executive Function (BRIEF) is a widely used assessment tool designed to evaluate EF behaviors in children and adolescents aged 5–18 years (Gui et al., 2022). This tool assesses EF behaviors in both home and school environments, providing a comprehensive overview of a child’s capabilities. The BRIEF comprises theoretically and statistically derived scales, which form two primary indexes: Behavioral Regulation and Metacognition. The Behavioral Regulation Index includes three scales (BRIEF_INHIBIT, BRIEF_SHIFT, BRIEF_EMOTIONAL), while the Metacognition Index includes five scales (BRIEF_INITIATE, BRIEF_WORKING MEMORY, BRIEF_PLAN/ORGANIZE, BRIEF_ ORGANIZATION OF MATERIALS, BRIEF_ MONITOR).

### Behavior assessment

The Social Responsiveness Scale (SRS) is a tool used to assess ASD features in children and adolescents (Chan et al., 2017). It provides a total score and five subscale scores that indicate the severity of ASD symptoms. The total score ranges from 0 to 59 for normal, 60 to 75 for mild abnormalities, 76 to 90 for moderate, and 91 or above for severe abnormalities. The five subscales assess areas such as social interaction, social communication, social awareness, social cognition, and repetitive behaviors (social manners), each with a score range of 0 to 39.

*T*-scores in SRS and BRIEF are calculated by converting raw scores into standardized scores, with a mean of 50 and a standard deviation of 10 (Nguyen et al., 2019). The interpretation of *T*-scores is as follows: a *T*-score of 50 indicates that the individual’s social responsiveness level is at the median of the normal range; a *T*-score above 60 typically indicates higher than average levels, which may suggest difficulties or autism traits; a *T*-score below 40 usually indicates lower than average social responsiveness levels, suggesting fewer issues in social interaction. *T*-scores were obtained from the ABIDE database.

### Data preprocesses

The resting-state fMRI data were pre-processed with a standardized pipeline using the Restplus V1.27 (Jia et al., 2019). Statistical Parametric Mapping (SPM12) was installed in MATLAB 2017b (Math Works, Natick, MA, United States). The processing pipeline included removing the first 10 repetition time (TR) images, slice-timing correction, motion realignment, spatial normalization to Montreal Neurological Institute (MNI) space, smoothing with a 6 mm^3^ Gaussian kernel, linear detrending, nuisance regression (for the following nuisance regressors: Friston’s 24 head-motion parameters, cerebrospinal fluid, white matter, and global signals), and temporal filtering (0.01–0.08 Hz). As the rsfMRI scanning duration varied between sites and the minimum was 5 min, we retained 5 min of the time course. This was done to ensure that the data from all participants remained consistent in terms of time series length, thereby avoiding the impact of time differences on the analysis results (Xie et al., 2022).

### ROI generation and definition of functional brain networks

**Figure 2:**
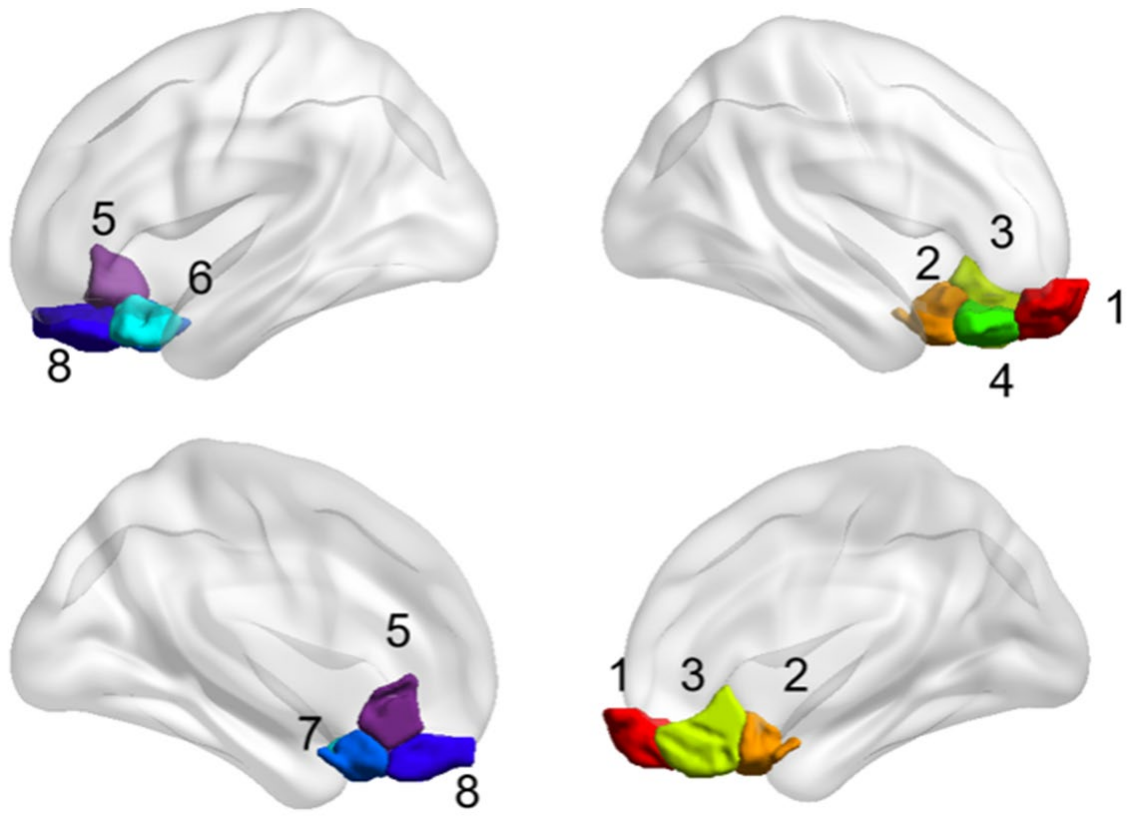
The brain map of the eight subregions of the orbitofrontal cortex.

The OFC and whole brain regions were chosen based on the Shen_268 mask (Shen et al., 2013). According to Yale atlas (Noble et al., 2017; Demuru et al., 2017), these functional regions form 10 brain networks, including the medial frontal network (MFN), the frontoparietal network (FPN), the default mode network (DMN), the motor network (MON), the visual I network (Vis I), the visual II network (Vis II), the visual association network (VA), limbic, basal ganglia, and cerebellum. The eight subregions of the OFC form different functional networks (Table 2 and Figure 2). Therefore, in this study, the eight subregions of the OFC were defined as eight seed, and the functional connectivity between these subregions and the whole brain was calculated.

**Table 2:** Information of eight subregions of orbitofrontal cortex.

	BA	MNI	Lobe	Network
1	OrbFrontal (11)	(13.86, 56.85, −16.64)	R-prefrontal	FPN
2	OrbFrontal (11)	(9.57, 17.75, −19.5)	R-prefrontal	LN
3	OrbFrontal (11)	(5.08, 34.9, −17.35)	R-prefrontal	DMN
4	OrbFrontal (11)	(15.63, 34.11, −22.59)	R-prefrontal	FPN
134	OrbFrontal (11)	(−5.42, 29.14, −10.12)	L-prefrontal	DMN
135	OrbFrontal (11)	(−18.22, 19.05, −20.98)	L-prefrontal	LN
136	OrbFrontal (11)	(−5.82, 18.18, −21.59)	L-prefrontal	LN
137	OrbFrontal (11)	(−8.15, 39.69, −21.44)	L-prefrontal	MFN

### Dynamic FC

Dynamic BC2.2 (Liao et al., 2014) was utilized to examine time-varying functional connectivity between eight OFC subregions and other brain areas. The sliding window method, a standard approach for quantifying time-varying functional connectivity, was employed (Savva et al., 2019). This method involves segmenting the data into multiple equal-length segments to enhance temporal resolution. As a guideline, the minimum window length should exceed 1/*f*_min_, where *f*_min_ is the lowest frequency of time courses (Leonardi and Van De Ville, 2015). Given a minimum TR value of 2 s in the dataset, we constructed functional connectivity matrix with a sliding window (rectangular time window) length of 30 TRs (60 s) and an overlap of 0.85, so if the first start time point is 1, the second window starts at 2.5 [floor (1 + (1 − 0.85) * 30)]. “Overlap” denotes the number of time points shared between consecutive windows. Within each window, we calculated the Pearson correlation analysis between eight OFC subregions and other brain areas. To assess time-varying functional connectivity, the variance matrix of functional connectivity in each window was analyzed for each participant.

### Statistical analysis

#### Differences in the variance of functional connectivity between the eight OFC subregions and other brain regions in ASD and TD individuals

ComBat harmonization was utilized to minimize site-related differences (Fortin et al., 2018). To maintain biologically relevant variability, we included age, group, mean FD, and IQ as covariates in the ComBat procedure. Then independent sample *t*-tests was used to assess the difference in the variance of functional connectivity between the ASD and TD groups (*p* < 0.05, FDR corrected), age and head movements were included as covariates to control for potential confounding factors. Performing statistical analysis using Graph Theoretical Network Analysis (GRETNA) toolbox (Wang et al., 2015).^1^ The tools Gretna and ComBat run in MATLAB 2017b.

#### Relationship among atypical variance, effective function and RRB

To identify the relationship between atypical variance, EF and RRB, linear correlation was used in IBM SPSS Statistics 27. Furthermore, we employed a linear regression analysis. Variance and the scores from each BRIEF component were used as independent variables, while SRS_MANNERS scores served as the dependent variable. Age and head movements were included as covariates to control for potential confounding factors.

#### Mediation effective of executive function between atypical variance and behavior

To investigate the mediating effect of EF on the relationship between atypical variance and behavior, we constructed a mediation model using Bootstrapping in IBM SPSS Amos 28. Variance were the independent variable, SRS_MANNERS were the dependent variable, and EF was the mediator. Perform bootstrap (number of bootstrap samples) is 2000. Bias corrected confidence intervals is 95.

## Results

### Demography

After quality control, a total of 203 participants were enrolled in this study, including 93 boys with ASD and 110 TDs. All the participants were males. There was no significant difference in age [*t* (202) =1.650, *p* = 0.101] between the children with ASD (mean age = 9.85 ± 2.4) and those with TDs (mean age = 10.09 ± 1.98). The detailed demographics and clinical information of the participants are shown in Table 3.

**Table 3:** Demographics and clinical information of the TDs and children with ASD.

Variable	*N*	ASD		*N*	TD		*t*	95% CI		*p*
		Mean	SD		Mean	SD		Lower	Upper	
Age [mean (SD)]	93	9.58	2.40	110	10.09	1.98	1.65	−0.10	1.11	0.10
ADI-R (C)	93	5.44	2.54	NA	NA	NA	NA	NA	NA	NA
IQ	93	112.10	17	110	114.80	14.38	1.15	−1.91	7.22	0.25
SRS_TOTAL	92	75.89	12.83	69	44.36	6.86	18.52	28.17	34.89	<0.01
SRS_AWARENESS	92	67.83	11.49	69	45.90	9.98	12.67	18.51	25.35	<0.01
SRS_COGNITION	92	71.98	12.28	69	43.01	6.33	17.89	25.77	32.16	<0.01
SRS_COMMUNICATION	92	73.87	13.91	69	44.67	6.90	16.01	25.60	32.80	<0.01
SRS_MOTIVATION	92	70.52	13.67	69	47.16	7.51	12.82	19.76	26.96	<0.01
SRS_MANNERISMS	92	77.12	16.43	69	45.72	7.11	14.86	9.98	16.33	<0.01
BRIEF_INHIBIT_T	83	58.96	11.36	68	45.81	7.53	8.19	16.42	23.88	<0.01
BRIEF_SHIFT_T	83	66.78	12.94	68	46.63	9.55	10.67	12.19	18.85	<0.01
BRIEF_EMOTIONAL_T	83	59.95	12.00	67	44.43	7.54	9.21	15.59	21.89	<0.01
BRIEF_BRI_T	83	62.96	11.30	67	44.22	7.25	11.75	10.92	17.20	<0.01
BRIEF_INITIATE_T	83	61.73	11.20	68	47.68	7.50	8.85	12.43	19.12	<0.01
BRIEF_WORKING_T	83	62.61	11.38	68	46.84	8.91	9.32	12.87	20.00	<0.01
BRIEF_PLAN_T	83	62.90	12.24	68	46.47	9.36	9.10	4.22	11.13	<0.01
BRIEF_ORGANIZATION_T	83	56.72	10.67	68	49.04	10.71	4.39	12.43	19.64	<0.01
BRIEF_MONITOR_T_1	82	61.96	11.09	68	45.93	11.17	8.79	13.37	19.94	<0.01
BRIEF_MI_T_1	83	63.31	10.70	68	46.66	9.46	10.02	15.67	22.08	<0.01
BRIEF_GEC_T_1	83	64.28	10.68	67	45.40	8.78	11.63	9.98	16.33	<0.01

### Difference in variance of functional connectivity between ASD and TD

**Figure 3:**
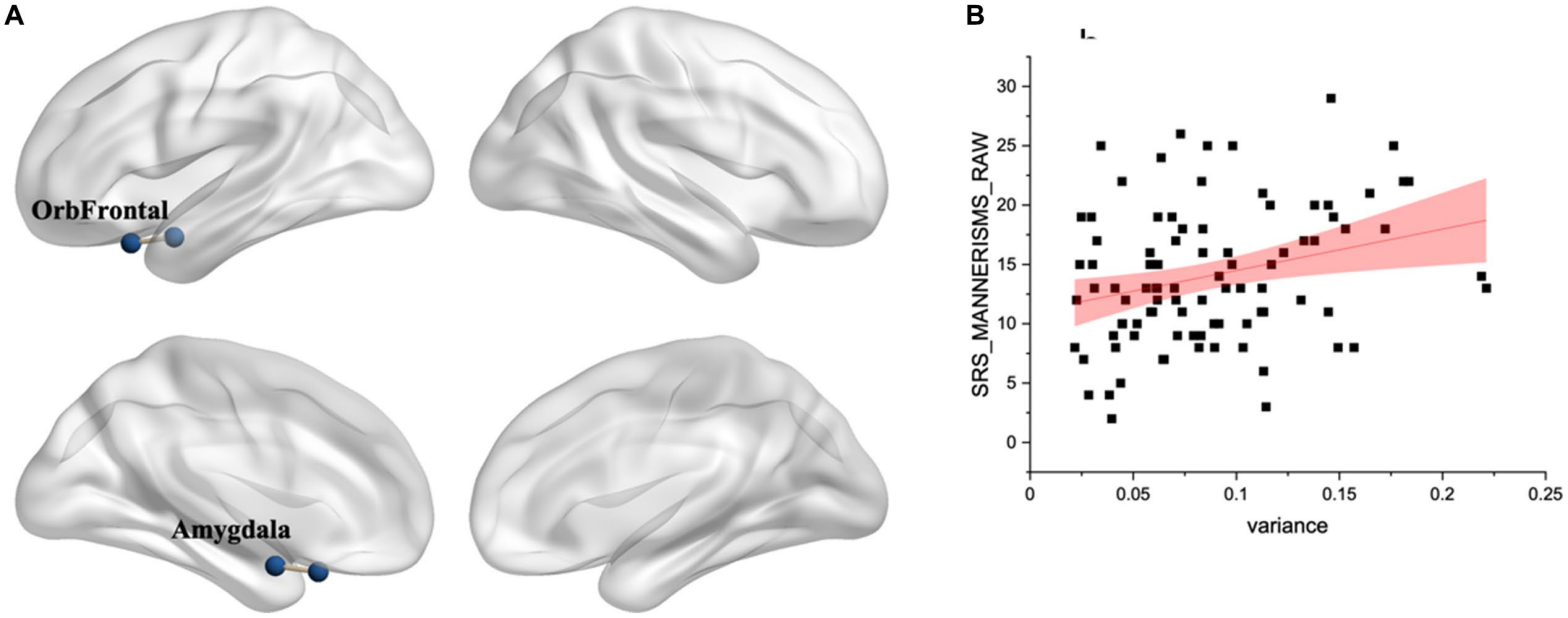
**(A)** Difference in variance of functional connectivity between ASD and TD. Significant differences identified in the L-prefrontal (limbic network) and left amygdala after FDR correction (*q* < 0.05). **(B)** There was a significant relationship with atypical variance and SRS_MANNER scores (*r* = 0.3, *p* < 0.01).

Comparing the time-varying characteristics of functional connectivity between the eight OFC subregions and other brain regions in ASD and TD groups, we found that, after FDR correction (q < 0.05), only the OFC subregion in the L-prefrontal (Limbic network) and the left amygdala (motor network) (Noble et al., 2017) showed significant differences (Figure 3A and Table 4).

**Table 4:** Difference in variance of functional connectivity between ASD and TD.

	MNI ordinate	*t*	FDR *q*	Lobe	Network
OrbFrontal	(−18.22, 19.05, −20.98)	5.00	<0.01	L-prefrontal	Limbic
Amygdala	(−26.79, 2.42, −18.71)			L-limbic	Motor

**Table 5:** Linear regression analysis of variance and executive functions on RRBs.

Model	Variable	df	Standardized *β*	*p*	Adjusted *R*^2^
1	Variance	142	0.15	0.02	0.46
	BRIEF_INHIBIT_T		0.62	<0.01	
2	Variance	141	0.19	<0.01	0.46
	BRIEF_EMOTIONAL_T		0.60	<0.01	
3	Variance	142	0.25	<0.01	0.18
	BRIEF_ORGANIZATION_T		0.26	<0.01	
4	Variance	141	0.15	0.02	0.48
	BRIEF_MONITOR_T		0.61	<0.01	

### Relationship among atypical variance, effective function and RRB

There was a significant relationship with variance and RRB (*r* = 0.3, *p* < 0.01) (Figure 3B). Additionally, there was a significant relationship between variance and EF, with correlations ranging from *r* = 0.18 to *r* = 0.33, *p* < 0.05. The regression analyses reveal that atypical and EF significantly impacts RRBs, controlling for age and mFD. Specifically, BRIEF_INHIBIT, BRIEF_EMOTIONAL, BRIEF_ORGANIZATION and BRIEF_MONITOR, with standardized *β* coefficients ranging from 0.60 to 0.62 and *p*-values all less than 0.01 (Table 5).

### Mediation effective of executive function between variance and behavior

**Figure 4:**
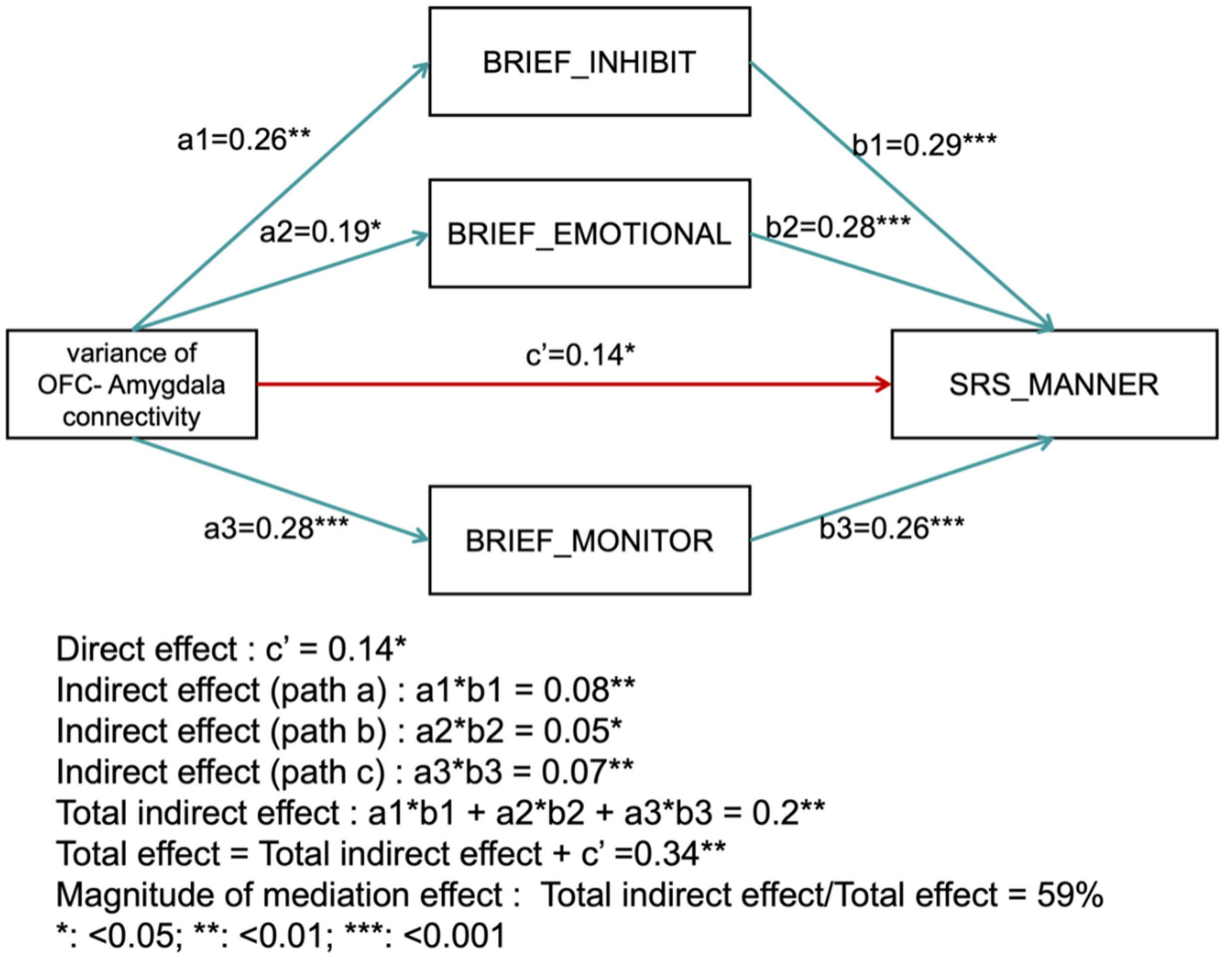
Mediation analysis results. The diagram illustrates the direct effect (*c*′) and indirect effects through mediators (BRIEF_INHIBIT, BRIEF_EMOTIONAL, BRIEF_MONITOR), as well as the total effect and the proportion of the mediation effect.

The mediation analysis assessed the impact of several mediators on the relationship between the independent variable and the dependent variable. The direct effect was found to be significant with a coefficient of *c*′ = 0.14 (*p* < 0.05). The indirect effects through the mediators were as follows: BRIEF_INHIBIT yielded an indirect effect of 0.08 (*p* < 0.01); BRIEF_EMOTIONAL contributed an indirect effect of 0.05 (*p* < 0.05); BRIEF_MONITOR produced an indirect effect of 0.07 (*p* < 0.01). In contrast, BRIEF_ORGANIZATION did not show a statistically significant indirect effect (*p* > 0.05). The total indirect effect, summing across all mediators, was 0.20 (*p* < 0.01). Including this total indirect effect with the direct effect resulted in an overall effect of 0.34 (*p* < 0.01). The magnitude of the mediation effect was calculated as the ratio of the total indirect effect to the total effect, yielding 59%. This indicates that the mediators—BRIEF_INHIBIT, BRIEF_EMOTIONAL, and BRIEF_MONITOR—account for 59% of the total effect (Figure 4).

## Discussion

This study found that executive functions in children with ASD were poorer than those in typically developing children. The increased dynamic characteristics of OFC-amygdala functional connectivity in children with ASD were associated with reduced executive functions. Furthermore, the increased dynamic characteristics of OFC-amygdala functional connectivity, reduced executive functions were related to heightened RRBs. Mediation analysis further revealed that executive functions (BRIEF_INHIBIT, BRIEF_EMOTIONAL, BRIEF_MONITOR) mediated the relationship between the dynamic characteristics of OFC-amygdala connectivity and RRBs.

We found that executive functions, as measured by the BRIEF subscales, were significantly poorer in children with ASD compared to TD children. This is consistent with previous research that highlights EF deficits, such as inhibitory control, emotional regulation, and self-monitoring, as hallmark impairments in ASD (Iversen and Lewis, 2021; Blijd-Hoogewys et al., 2014). In addition to confirming findings from previous studies regarding reduced emotional regulation abilities of individuals with ASD, we further discovered that this reduction is associated with an increase in the dynamic features of OFC (in LN)-amygdala connectivity. The significantly higher variance in functional connectivity between the OFC and amygdala in the ASD group compared to the TD group suggests that individuals with ASD experience greater fluctuations in neural communication within this circuitry (Fu et al., 2021). The OFC and amygdala play critical roles in emotional regulation and decision-making (Gao et al., 2016; Zheng et al., 2018; Li et al., 2021). The OFC, particularly within the limbic network (LN) (Noble et al., 2022), is involved in monitoring and evaluating emotional experiences to guide goal-directed behavior (Rolls et al., 2020), while the amygdala is responsible for generating and regulating emotions, including managing anxiety (Wang et al., 2013). The projections from the OFC to the amygdala have been identified through both optogenetics (Malvaez et al., 2019) and fiber photometry (Li et al., 2021), and these projections are excitatory and glutamatergic. Different subregions of the OFC project differently: the medial OFC (mOFC) is more closely connected to the basolateral amygdala (BLA), while the lateral OFC (lOFC) is more likely to be related to the lateral amygdala (Malvaez et al., 2019). Given the key roles these brain regions play in emotional regulation, the increased dynamic features of OFC (in LN)-amygdala connectivity, reflecting heightened sensitivity or instability in this circuitry, are likely to contribute to poor emotional regulation in children with ASD.

The OFC-amygdala projection matures gradually around puberty and plays a crucial role in regulating social behavior, fear memory, and emotional responses (Li et al., 2021). Abnormal developmental patterns are associated with various neurodevelopmental disorders (Andrews et al., 2022; Hessl et al., 2021; Xu et al., 2020). For instance, in individuals with autism, the amygdala is larger during childhood but shows slower growth, which correlates with differential changes in anxiety related to traditional DSM anxiety and autism-related anxiety (Andrews et al., 2022). Furthermore, mouse models with KMT2E gene haploinsufficiency show alterations in the number and size of amygdala neurons, which may be a potential mechanism for the social deficits observed in autism (Li et al., 2023). Morphological MRI studies report that children and adolescents with autism have reduced total right OFC volume (i.e., gray matter plus white matter), whereas adults show an increase in right OFC volume (Li et al., 2023). This highlights the importance of studying this circuit around puberty.

In addition, we found that the increased dynamic characteristics of OFC-amygdala connectivity were associated with an increase in RRBs. The association between increased OFC-amygdala connectivity variance and more pronounced RRBs further highlights the potential impact of neural instability on behavioral manifestations of ASD (Dinstein et al., 2015). Similar to previous research, our study found that increased time-varying characteristics of functional connectivity are positively correlated with ASD symptom severity (Li et al., 2020). The findings suggest that these behaviors could be exacerbated by the fluctuating connectivity patterns between key brain regions involved in executive functions.

We also found that the decline in executive functions was associated with an increase in RRBs. A decrease in emotional control ability was related to increased RRBs, further supporting the hypothesis that atypical changes in the OFC-amygdala circuitry are linked to emotional regulation abnormalities and may contribute to the development of RRBs (Iversen and Lewis, 2021). Inhibitory control deficits are commonly observed in individuals with ASD and are associated with RRBs (Schmitt et al., 2018). Inadequate inhibitory control can contribute to impulsive behaviors that disregard social norms (Diamond, 2013). Self-monitoring, on the other hand, reflects our ability to understand and adjust our behaviors based on our emotional states (Dorman et al., 2019). This function helps individuals recognize and manage their emotional responses, such as using techniques like deep breathing or cognitive reappraisal to alleviate anxiety.

The mediation analysis offers a deeper understanding of how these neural fluctuations might translate into RRBs (Lee et al., 2019). Specifically, increased variance in functional connectivity between the OFC and amygdala may disrupt inhibitory control, impairs emotional regulation, and reduces monitoring ability, leading to difficulties in stopping or managing behaviors, reducing anxiety and stress, and behavioral adjustment (Koch et al., 2018). These EFs dysregulation, in turn, exacerbates RRBs as a coping strategy (Wigham et al., 2015). While, previous research has highlighted the role of executive dysfunction in ASD behaviors (Jones et al., 2018; Cai et al., 2018), our results provide support for the hypothesis that instability in the OFC-amygdala connectivity may impair EFs, which are crucial for RRBs (Lindsay and Creswell, 2019; O’Malley et al., 2024).

## Conclusion

By establishing a direct link between increased connectivity variance, EF impairment, and RRBs, our study suggests that therapeutic interventions targeting neural connectivity stability could be beneficial. Enhancing EF and improving emotional regulation might mitigate the negative effects of increased connectivity variance, potentially reducing RRBs and improving overall behavioral outcomes in individuals with ASD (Liang et al., 2022). Further research is needed to explore interventions that can stabilize neural connectivity and strengthen EFs in this population.

### Limitation

One limitation of this study is the cross-sectional design, which does not allow for causal inference regarding the relationship between executive functions, OFC-amygdala connectivity, and RRBs in children with ASD. Longitudinal studies are needed to better understand the temporal dynamics of these relationships and to explore potential causal pathways. Additionally, the participants in this study were all from Western countries, which may limit the generalizability of the findings to populations from other cultural or geographical regions. Future research should consider a more diverse sample to enhance the external validity of these results.

## Data Availability

Publicly available datasets were analyzed in this study. This data can be found here: the imaging data for this study are available from the Autism Brain Imaging Data Exchange (ABIDE) II database (https://fcon_1000.projects.nitrc.org/indi/abide/abide_II.html).

## Footnotes

^1^ http://www.nitrc.org/projects/gretna/
